# Maturation and shuttling of the yeast telomerase RNP: assembling something new using recycled parts

**DOI:** 10.1007/s00294-021-01210-2

**Published:** 2021-09-02

**Authors:** Louise Bartle, Yulia Vasianovich, Raymund J. Wellinger

**Affiliations:** 1 Department of Microbiology and Infectious Diseases, Faculty of Medicine and Health Sciences, Université de Sherbrooke, Applied Cancer Research Pavilion, 3201 rue Jean-Mignault, Sherbrooke, QC J1E 4K8 Canada; 2 Department of Medicine, McGill University Health Center, McGill University, 1001 Blvd. Décarie, Montreal, QC H4A 3J1 Canada

**Keywords:** Telomere stability, Telomerase, RNA processing, RNA trafficking, RNA maturation, Telomerase assembly

## Abstract

As the limiting component of the budding yeast telomerase, the Tlc1 RNA must undergo multiple consecutive modifications and rigorous quality checks throughout its lifecycle. These steps will ensure that only correctly processed and matured molecules are assembled into telomerase complexes that subsequently act at telomeres. The complex pathway of Tlc1 RNA maturation, involving 5'- and 3'-end processing, stabilisation and assembly with the protein subunits, requires at least one nucleo-cytoplasmic passage. Furthermore, it appears that the pathway is tightly coordinated with the association of various and changing proteins, including the export factor Xpo1, the Mex67/Mtr2 complex, the Kap122 importin, the Sm_7_ ring and possibly the CBC and TREX-1 complexes. Although many of these maturation processes also affect other RNA species, the Tlc1 RNA exploits them in a new combination and, therefore, ultimately follows its own and unique pathway. In this review, we highlight recent new insights in maturation and subcellular shuttling of the budding yeast telomerase RNA and discuss how these events may be fine-tuned by the biochemical characteristics of the varying processing and transport factors as well as the final telomerase components. Finally, we indicate outstanding questions that we feel are important to be addressed for a complete understanding of the telomerase RNA lifecycle and that could have implications for the human telomerase as well.

## Introduction

Telomeres, the protective caps located on the ends of eukaryotic chromosomes, play a vital role in shielding chromosomes from erosion, DNA loss and end-to-end fusions that all could cause massive genome rearrangements. In human somatic cells, telomeric DNA shortens after each replication cycle due to the inability of the replication machinery to entirely duplicate the lagging DNA strand of chromosome ends, and due to the 5' DNA end resection activities at the leading chromosome ends that are required for reconstitution of the telomeric 3'-overhangs (Wellinger et al. 1996; reviewed in Lingner et al. 1995; Wellinger 2014). In germ, stem and cancer cells, as well as in many unicellular eukaryotes such as ciliates and yeasts, telomeric DNA is maintained by the telomerase RiboNucleoProtein (RNP) complex that is a specialised reverse transcriptase. Telomere extension by telomerase involves addition of telomeric repeats onto the 3' end of the G-rich strand (Greider and Blackburn 1985; Greider and Blackburn 1987; Singer and Gottschling 1994; Wellinger and Zakian 2012), while the C-rich strand will be replenished by continued lagging strand synthesis (Dionne and Wellinger 1998; Diede and Gottschling 1999; Martin et al. 2000; Parenteau and Wellinger 2002; Wellinger and Zakian 2012). In *Saccharomyces cerevisiae*, telomerase extends the 3’ telomere end using a template sequence that is located within the long non-coding telomerase RNA called TeLomerase Component 1 (Tlc1 RNA, Singer and Gottschling 1994). Given that the Tlc1 RNA is the limiting component of the yeast telomerase (Mozdy and Cech 2006; Bajon et al. 2015), its biogenesis pathway is critical for the function of the entire telomerase RNP. Therefore, the understanding of Tlc1 processing and maturation has been investigated heavily ever since its initial discovery over 25 years ago (Singer and Gottschling 1994). Indeed, Tlc1 undergoes processing and trafficking steps before reaching maturation and being able to function on the telomerase substrate, the telomeres. Curiously, nucleo-cytoplasmic RNA shuttling, some specific RNA attributes, such as 5'- and 3'-end modifications, as well as certain protein associations required for RNA stability and maturation, appear to be shared between Tlc1 and a variety of RNAs that belong to very different classes such as small nuclear RNAs (snRNAs), small nucleolar RNAs (snoRNAs) and messenger RNAs (mRNAs). However, ultimately, the Tlc1 RNA follows a unique maturation pathway, and thus, constitutes a separate RNA niche. The core and, in many species, the highly conserved function of the telomerase RNP is to provide the RNA template for reverse transcription. However, different species utilise a wide variety of RNA stabilising mechanisms and maturation pathways (reviewed in Podlevsky and Chen 2016). Here, we will focus on the unique biogenesis pathway of the budding yeast telomerase RNP with special attention to its RNA component and exciting recent discoveries.

## Tlc1: a unique transcript

**Fig. 1:**
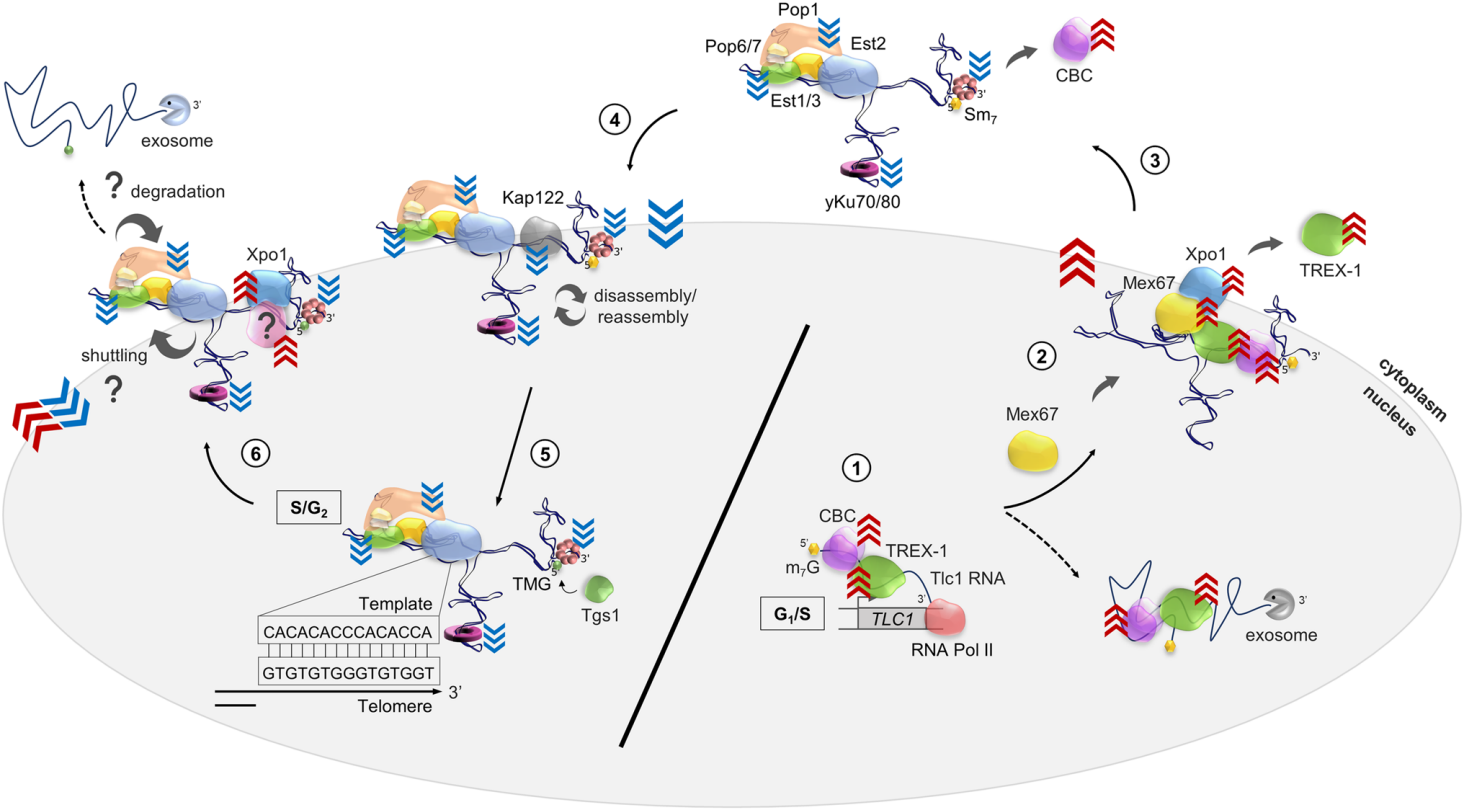
Model of the telomerase RNA biogenesis in budding yeast. (1) The Tlc1 RNA is transcribed in G1/S phase by RNA polymerase II. The newly transcribed RNA presumably is m^7^G-capped at the 5’-end and most likely is co-transcriptionally bound by the CBC and TREX-1 complexes. (2) Mex67 binds to the Tlc1 RNA through TREX-1 or other proteins and serves as an adaptor for the Xpo1 export factor. Mex67 association ensures an intranuclear stability of the newly transcribed Tlc1 RNA and protects it from degradation by the nuclear exosome (stippled arrow). The Tlc1 RNA bound by CBC, Mex67 and Xpo1 acquires unique biochemical properties that force it out of the nucleus (indicated by red chevrons). (3) Once on the cytoplamsic side, the RNP dissociates from the Mex67–Xpo1 proteins and may also lose the TREX-1 complex. In addition, binding of the Sm_7_ complex ensures stability of the Tlc1 RNA. Sm_7_ may also facilitate dissociation of CBC, which may otherwise direct the RNA for translation/degradation. The telomerase protein subunits Pop1, Pop6, Pop7, Est1, Est2, Est3 and yKu70/80 likely associate with Tlc1 in the cytoplasm, although binding of these components prior to the cytoplasmic export or after the nuclear re-import is also possible. (4) Telomerase proteins bound to Tlc1 contain nuclear localisation signals which change the biochemical properties of the RNP forcing it into the nucleus via the Kap122-mediated import pathway (indicated by blue chevrons). (5) The re-imported telomerase RNP undergoes nucleolar 5’-TMG capping mediated by Tgs1. In late S/G2 phase, the mature RNP elongates 3’-telomere ends using the template of the Tlc1 RNA. Disassembly and reassembly of the telomerase RNP (indicated by curved arrows) likely takes place during the telomerase lifecycle which may serve as a mechanism regulating its timely action at telomeres. (6) Tlc1 could possibly undergo additional rounds of nucleo-cytoplasmic shuttling (indicated by curved arrows and question mark). In this scenario, an unknown adaptor other than Mex67 (indicated by a question mark) will be required for the Xpo1-dependent RNA export and a new set of proteins will force Tlc1 into the cytoplasm (indicated by red chevrons). Tlc1 is eventually degraded by unknown mechanisms (indicated by question mark), with degradation potentially occurring at any time when Tlc1 is misfolded

The Tlc1 RNA is a 1,158 nt RNA polymerase II transcript of very low abundance (10–25 molecules per cell) (Dandjinou et al. 2004; Mozdy and Cech 2006; Bajon et al. 2015). The low transcription of the RNA is cell cycle regulated, peaking at the G1-S transition (Fig. 1, step 1; Dionne et al. 2013). A minor fraction (5–10%) of the Tlc1 transcripts are polyadenylated (poly-A+) after transcription (Chapon et al. 1997), but the majority undergo transcriptional termination and processing via the Nrd1-dependent snRNA pathway to form poly-A- Tlc1 (Jamonnak et al. 2011; Noël et al. 2012). As a result, both poly-A+ and poly-A- Tlc1 species are detectable in growing *S. cerevisiae* cells (Chapon et al. 1997; Ferrezuelo et al. 2002; Noël et al. 2012). However, it remains unclear as to why a significant fraction of the Tlc1 RNA is produced as a poly-A+ form, since this form does not contribute to, nor is found, in active telomerase (Bosoy et al. 2003; Noël et al. 2012). Additonally, modifications near the 3’-end of the *TLC1* gene that prevent the formation of the poly-A+ Tlc1 RNA have no detectable effect on telomerase function either (Noël et al. 2012). There is evidence to suggest that the rare poly-A+ Tlc1 can undergo processing via an alternative pathway to form mature poly-A- Tlc1 (Chapon et al. 1997; Bosoy et al. 2003; Noël et al. 2012). However, given the most likely non-essential nature of the rare poly-A+ form, details of this pathway for Tlc1 generation are scarce and it will not be discussed further.

## After *TLC1* transcription: stabilise the ends and get out of the nucleus

The Tlc1 RNA is one of the most stable RNAs in budding yeast cells. Compared to mRNAs with an average turnover of only 4.8 min, Tlc1 has a remarkably long half-life of at least 60 min (Larose et al. 2007; Chan et al. 2018). Due to limitations of current experimental approaches, a precise life-span of the telomerase RNA is yet to be determined. In fact, the Tlc1 lifecycle could be much longer than estimated and approach the extreme stability of snRNAs which can persist in cells for several hours (Stutz et al. 1993). Therefore, the available estimate suggests exquisite stabilisation mechanisms for the Tlc1 RNA. Yet we are only just beginning to understand what specific events or factors ensure such prominent stability of the telomerase RNA.

It is assumed that like virtually all other RNA polymerase II transcripts, the Tlc1 RNA initially harbours a 7-methylguanylate (m^7^G) cap at its 5'-end (Fig. 1, step 1). Also, akin to the other RNAs, this initial cap most likely is added in a co-transcriptional fashion, but as of yet, for Tlc1 there is no direct experimental demonstration of this. When thinking of maturation events after transcription, it is useful to consider the pathways ascribed to related transcripts. On both snRNAs and mRNAs, the m^7^G cap is bound by the Cap-Binding Complex (CBC) that in yeast is comprised of Sto1 and Cbc2 (Colot et al. 1996). CBC can interact with Mex67/Mtr2 and Xpo1, factors that facilitate the passage of snRNAs and mRNAs through the nuclear pores to the cytoplasm (Fig. 1, step 2; Hamm and Mattaj 1990; Izaurralde et al. 1995). While it is reasonable to propose that Tlc1 RNA also binds CBC prior to nuclear export, this has yet to be determined. The Xpo1(CRM1) export factor was shown to be implicated in Tlc1 transport from the nucleus to the cytoplasm (Gallardo et al. 2008) and a recent study examining the nuclear export specifically of newly synthesised Tlc1 revealed that indeed this process is mediated exclusively by Xpo1 (Fig. 1, step 2; Vasianovich et al. 2020). However, and quite unexpectedly, Mex67/Mtr2 was found to be crucial for the 3'-end stability of newly transcribed Tlc1, even prior to its nuclear export and not necessarily just for export itself (Vasianovich et al. 2020). Indeed, in the absence of Mex67, new Tlc1 transcripts undergo rapid Rrp6-dependent degradation by the nuclear exosome. This finding mirrors earlier data showing that in cells harbouring a *mex67* mutation, newly synthesised mRNAs are also rapidly degraded before nuclear export (Tudek et al. 2018). However, in that study, the intranuclear instability of mRNAs in part was caused by exhaustion of the nuclear poly-A binding protein Nab2 which was a consequence of the mRNA export block (Tudek et al. 2018). Given that Tlc1 RNA is a poly-A- RNA and presumably not bound by Nab2, its nuclear instability may be attributed to something other than Nab2 exhaustion. In support of this, in cells with a temperature-sensitive *xpo1-1* mutation and in which newly synthesised mRNAs and Tlc1 RNA accumulate in the nucleus, the Tlc1 RNA is stable (Vasianovich et al. 2020). Hence, these data reinforce the idea that newly transcribed Tlc1 RNAs rely on the analogous Xpo1-mediated export pathway that is used for m^7^G- and CBC-capped RNAs. Additional to this, the new study implies that the Tlc1 RNA requires a stabilising activity mediated by Mex67/Mtr2 prior to export.

At present, it is not known how the Mex67/Mtr2 complex mediates Tlc1 stability and protects it from degradation by the nuclear exosome. In terms of the Mex67/Mtr2 function on mRNAs and their transport, it is known that during mRNA transcription, an evolutionarily conserved Transcript–EXport 1 (TREX-1) complex, comprised of THO subunits (Mft1, Hpr1, Tho2, Thp2, Tex1), Yra1 and Sub2, associates with mRNAs in a CBC-dependent manner (Peña et al. 2012; reviewed in Okamura et al. 2015). This association plays a crucial role in coordination of continued mRNA transcription, maturation, quality control and finally the binding of the Mex67/Mtr2 complex that is thought to mediate export to the cytoplasm. Whether this suite of associations holds for the Tlc1 RNA remains to be seen, but it is worth considering that Xpo1, the exclusive export factor for Tlc1, does not directly bind RNA (Hutten and Kehlenbach 2007). Therefore, Xpo1 requires an adaptor protein as a bridge to the RNAs to be exported. In metazoa, the phosphorylated adaptor for RNA export (PHAX) connects Xpo1(CRM1) with telomerase RNA and other RNA types (Ohno et al. 2000; Boulon et al. 2004). However, for yeast, a clear homologue of PHAX is not known. Given the strong indications implicating Mex67/Mtr2 for this role, we speculate that this complex in fact could be the Tlc1 adaptor for Xpo1, acting as a link between the RNA and the export receptor and thus ensuring RNA stability as well as transport (Fig. 1, step 2). Therefore, Mex67/Mtr2 may be viewed not just as a mobile nucleoporin, but rather as an RNA chaperone involved in a variety of intranuclear processes including RNA stabilisation and nucleo-cytoplasmic shuttling. It is also worth considering that binding of TREX-1 components or other proteins on Tlc1 may occur prior to Mex67/Mtr2 binding. Taken together, our hypotheses put forward here explain how in Mex67-deficient cells, mRNAs as well as the Tlc1 RNA manage to be transcribed and generated but cannot be channelled for export and instead are handed over to the nuclear exosome for degradation.

It has long been known that the 3'-end of the mature Tlc1 RNA is bound by the Sm_7_ heptameric protein complex (Seto et al. 1999). This so-called Sm_7_ ring binds to selected RNAs, in particular snRNAs, that contain a specific Sm_7_-binding motif near their 3'-end (Jones and Guthrie 1990). A long-standing model for Tlc1 3'-end protection suggests that the Sm_7_ complex associates with Tlc1 rapidly after transcription (Gallardo et al. 2008), thereby preventing intranuclear 3'-end degradation. However, and very much surprisingly, recent analyses specifically of newly transcribed Tlc1 molecules showed that prior to export to the cytoplasm, Sm_7_ binding was not required for Tlc1 stability (Vasianovich et al. 2020). Indeed, Tlc1 molecules engineered to lack the Sm_7_-binding site and thus without the ability to bind the Sm_7_ ring are transcribed and do reach the cytoplasm, but then get degraded there (Vasianovich et al. 2020). Hence, the Sm_7_ ring only becomes essential for Tlc1 stability once the RNA reaches the cytoplasm (Fig. 1, step 3). Moreover, the very few detectable Sm_7_-lacking molecules appeared unable to re-enter the nucleus, implicating Sm_7_ ring binding in mediating telomerase re-import as well (see below; Vasianovich et al. 2020). In a situation where Sm_7_-lacking Tlc1 RNAs are expressed and nuclear export is prevented, the RNA remains in the nucleus and is stable (Vasianovich et al. 2020). Hence, Tlc1 stability during the intranuclear phase right after transcription is not dependent on Sm_7_ ring binding. These findings conceptually are similar to those described for the maturation pathway of snRNAs, for which the Sm_7_ ring is required for stability only after export to the cytoplasm, not in the nucleus prior to export (Becker et al. 2019). Nevertheless, and as mentioned above, for newly transcribed Tlc1 RNA molecules, the intranuclear stability prior to export is very much dependent on Mex67/Mtr2. Remarkably, the in vivo labelling system further revealed that fully matured Tlc1 molecules that had completed one round of shuttling and returned to the nucleus (presumably with the Sm_7_ ring bound near the 3'-end) were not sensitive to a loss of Mex67/Mtr2 function anymore (Fig. 1, step 6). This is in line with the chaperoning role of Mex67/Mtr2 proposed above, which will only direct new RNA transcripts towards nuclear export but may not be required for re-imported RNAs. Therefore, 3’-end protection of the Tlc1 RNA occurs in distinct phases requiring different protein associations: Mex67/Mtr2 ensures telomerase RNA stability before its nuclear export, whereas Sm_7_ takes over Tlc1 3’-end protection following its cytoplasmic export and subsequent nuclear re-entry. In which cellular sub-compartment exactly the Sm_7_ ring associates with the Tlc1 RNA still remains to be determined. Even though Sm_7_ stabilises Tlc1 only after its nuclear export, it is still possible that Sm_7_ binding occurs in the nucleus directly after Tlc1 transcription. Alternatively, the Sm_7_ complex could associate with and stabilise Tlc1 only in the cytoplasm, similar to what was shown for snRNA (Becker et al. 2019). It is worth noting that knocking out Sm_7_ proteins in human cells results in cytoplasmic snRNA 3’-oligouridylation and subsequent degradation by either DIS3L2 or LSm/XRN1 (Roithová et al. 2020). Therefore, while the nucleases involved in cytoplasmic Tlc1 degradation remain undetermined, Tlc1 molecules that completely lack the Sm_7_ ring could be tested for 3'-oligouridylation as a useful first step.

For mRNAs, once they are in the cytoplasm, the nuclear CBC is replaced by the translation initiation factor eIF4F that binds to the cap and aids in recruitment of mRNAs to the ribosomes for translation (Gingras et al. 1999; Fortes et al. 2000). For snRNAs, CBC is thought to dissociate in the cytoplasm. For example, in higher eukaryotes, CBC contributes to snRNA nuclear export and subsequently in the cytoplasm, it is displaced by importin-β which has strong binding affinity within the nuclear pore complex, driving nuclear re-import (Izaurralde et al. 1995; Görlich et al. 1996; Palacios et al. 1997; Huber et al. 2002; Dias et al. 2009; Stanley et al. 2018). Additionally, it has been noted that several snRNA structures show the actual 5’- and 3’-ends to be in close spatial proximity (Hughes et al. 1987; Kretzner et al. 1990; Wan et al. 2016) and one could speculate that Sm_7_ ring binding at the 3'-end may cause CBC dissociation from the 5'-end and/or not allowing for association of eIF4F. The 3'- and 5'-ends of Tlc1 RNA are also in close spatial proximity (Dandjinou et al. 2004; Zappulla and Cech 2004). Therefore, the snRNA-like arrangement of Tlc1 with the juxtaposition of the Sm_7_ ring at the 3'-end and the 5'-end may disfavour CBC binding as reported for most snRNAs (Schwer et al. 2011). This would also protect the RNA from being directed towards ribosomes which, given the absence of a canonical open reading frame (ORF), would lead to subsequent degradation (Fig. 1, step 3; reviewed in Amrani et al. 2006).

## Completing the first shuttling cycle: returning to the nucleus, passing through the nucleolus

Given the nuclear telomere localisation, the Tlc1 RNA, in one form or another, has to get back into the nucleus. A limited screen of potential re-importer candidates suggested Kap122 as a Tlc1 re-import factor (Fig. 1, step 4; Gallardo et al. 2008). However, Kap122 is not essential and cells lacking this protein have only a mild, if any, telomeric phenotype (Vasianovich et al. 2020). Therefore, other and so far unknown transporters must be involved in Tlc1 re-import from the cytoplasm. Whether or not Mtr10 works at this step in the Tlc1 maturation pathway remains to be determined. Early results were suggestive of such a possibility (Ferrezuelo et al. 2002), but given the strong pleiotropic phenotypes of an *MTR10* deletion, the mechanistic role of Mtr10 remains nebulous.

For most species, during snRNA maturation, the bound Sm_7_ ring not only promotes 3'-RNA stabilisation but also aids in increased affinity of the Tgs1 enzyme for 5’-end hypermethylation (addition of trimethyl-guanosine at the 5'-end, abbreviated as TMG) (Plessel et al. 1994; Matera and Wang 2014; Becker et al. 2019). In human, plant, and animal cells, the presence of Cajal bodies within the nucleolus provides a concentration of proteins and a place for snRNA accumulation and methylation by Tgs1. Even though Cajal bodies have not been detected in yeast, there is evidence of a similar purposeful nucleolar Tgs1 localisation (Verheggen et al. 2002; Cioce and Lamond 2005), supporting the notion that in yeast, snRNAs and snoRNAs undergo trimethylation in the nucleolus. This final maturation step of the 5'-end, therefore, requires nuclear re-entry of the RNAs followed by a passage through the nucleolus. This progression from Sm_7_ binding, nuclear re-entry, to 5'-hypermethylation in the nucleolus most likely also applies to the Tlc1 RNA (Vasianovich et al. 2020). Formation of the TMG cap on Tlc1 completes the 5'- and 3'-end modification and maturation steps of the RNA (Fig. 1, step 5).

As mentioned above, in the absence of Sm_7_ binding at the 3'-end, the Tlc1 RNA is degraded and lost in the cytoplasm. This underscores the functional relevance of a proper 3'-end maturation. On the other hand, in the absence of Tgs1 and the 5'-TMG cap, only a slight nuclear Tlc1 enrichment is observed (Gallardo et al. 2008), but no significant change in overall Tlc1 abundance or any major telomeric phenotypes are caused (Franke et al. 2008). Except for the U1 snRNP, an absence of the TMG cap does not affect composition or function of the snRNPs either (Schwer et al. 2011). It is, therefore, unclear if the TMG cap on the Tlc1 RNA has any role in telomerase function or whether it is just a by-product of the 3'-end stabilisation mechanism triggered by the Sm_7_ complex. Notably, a lack of telomerase RNA methylation by Tgs1 homologs in species other than budding yeast have diverse and somewhat opposite consequences. For example, in human cells, a loss of TGS1 leads to increased telomerase RNA levels and telomere lengthening (Chen et al. 2020), while in *Schizosaccharomyces pombe,* the telomerase RNA levels decrease (Tang et al. 2012). Nevertheless, in wild type budding yeast, active telomerase appears to contain only TMG-capped Tlc1 RNAs (Seto et al. 1999; Bosoy et al. 2003). This finding allows consideration of two possibilities with respect to TMG capping. First, the telomerase RNP is only fully assembled after TMG capping in the nucleus. This possibility would allow for quality control of the RNA and elimination of faulty molecules before association of the other telomerase RNP proteins. Second, the telomerase RNP is assembled before TMG capping, most likely in the cytoplasm before re-import, essentially still harbouring the m^7^G cap. This idea would mean that re-import of Tlc1 and hypermethylation of its 5'-end occurs in the context of the whole RNP, as well as being an obligatory step prior to its entry into the nucleoplasm and subsequent use at telomeres. Therefore, further work is required to fully understand the mechanistic details and the role of TMG capping in Tlc1 maturation in budding yeast.

## Additional rounds of Tlc1 shuttling

The above describes the solid evidence for at least one round of Tlc1 RNA shuttling from the nucleus to the cytoplasm and back. At present, it is unknown whether this cycle repeats and, if it does, how many times. If Tlc1 shuttles multiple times, why would it do so? Theoretically, the cycles of Tlc1 trafficking in and out of the nucleus could be used as checkpoints to ensure it remains properly folded and functional. This is especially important for Tlc1, as its stability and long lifecycle potentially allow for it to be used multiple times as a template for telomeric repeat synthesis. In fact, there is some evidence that Tlc1 shuttles more than one time. First, an analysis of the subcellular localisation specifically of matured Tlc1 RNAs in wild type cells over time shows no late accumulation of those molecules in the nucleus, as would be expected if there was only one round of shuttling (Vasianovich et al. 2020). Second, even in cells with a conditional heat-sensitive mutation in *MEX67* (that encodes the RNA chaperone Mex67, see above), in which after a temperature shift virtually no new Tlc1 molecules are generated*,* the nuclear vs cytoplasmic distribution of the stable pre-existing Tlc1 molecules remains normal. Therefore, these molecules appear to continuously shuttle in and out of the nucleus even in the absence of a functional Mex67 protein (Vasianovich et al. 2020).

As outlined above, the nuclear Tlc1 RNA just off transcription may not have a Sm_7_ ring at its 3’-end, but its stability is ensured by Mex67. After the first round of shuttling, the nuclear RNA contains a Sm_7_ ring bound at the 3'-end. Furthermore, the freshly made Tlc1 RNA has an m^7^G cap at its 5'-end while the one after the first cycle contains a TMG cap. In addition, if the CBC complex associates with the m^7^G cap of newly synthesised Tlc1, most likely it will be removed in the cytoplasm prior to nuclear re-import and nucleolar TMG capping. Therefore, the RNP generated right after transcription is different from the one that has completed the first shuttling cycle (Fig. 1). By extension, this may mean that after the initial round of shuttling, the re-export of Tlc1 requires other factors than the first time around (Vasianovich et al. 2020). Consistent with this, Mex67 seems to be dispensable for the re-export of mature Tlc1. Moreover, in the absence of CBC, mature Tlc1 will require alternative RNA-binding proteins for Xpo1-mediated export or a completely different, Xpo1-independent export system (Fig. 1, step 6). There is precedence for such differential requirements of shuttling proteins in the RNA world. The transfer RNA^Phe^ (tRNA^Phe^) molecules require Mex67/Mtr2, Xpo1 (CRM1), Los1 and Msn5 for the first round of nuclear export, while only requiring Mex67/Mtr2 and Xpo1 (CRM1), but neither Los1 or Msn5 for a subsequent second re-export (Nostramo and Hopper 2020). This agrees with another report showing that tRNA export proteins have differential preferences for various tRNAs, and that Mex67/Mtr2-Los1 is utilised by a subset of tRNAs only for the initial nuclear export (Chatterjee et al. 2017). Therefore, a differential requirement for export proteins for newly synthesised versus mature Tlc1 molecules that have completed a first round of shuttling is quite likely. However, these details of the Tlc1 lifecycle remain to be elucidated.

Taken together, evidently, while it is reasonable to assume that Tlc1 undergoes multiple rounds of shuttling, to date, all work relies on experimental approaches using large populations of cells and/or snapshots in time. Live cell single-molecule tracking could unambiguously document such additional rounds and also allow investigations on the genetic requirements for the two different types of shuttling events.

## Tlc1 association with telomerase proteins and recruitment to telomeres

**Fig. 2:**
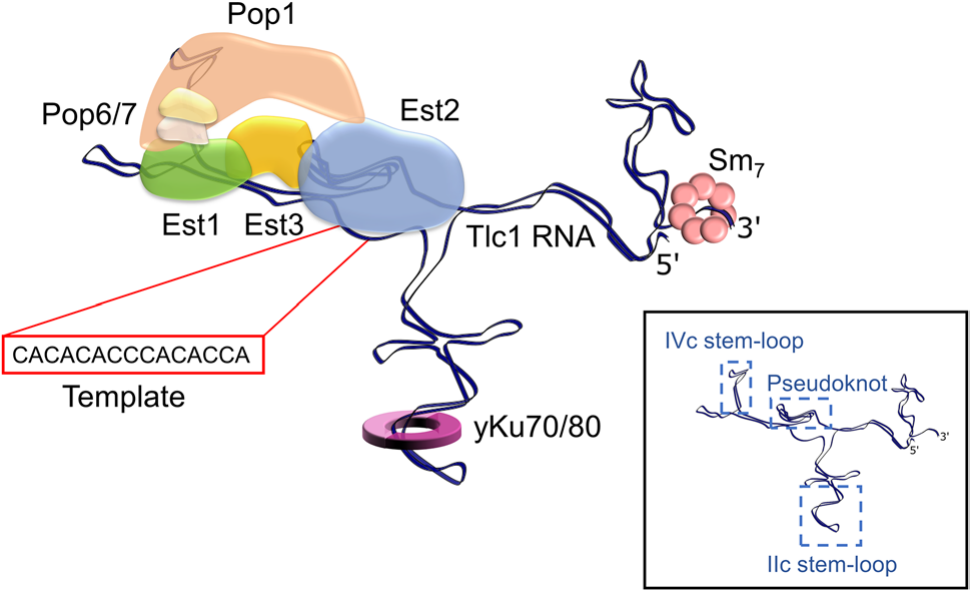
Schematic of the Tlc1 binding locations of Pop1, Pop6, Pop7, Est1, Est2, Est3, yKu70/80 and Sm_7_. The Pop proteins and Est1 bind to the IVc stem-loop. The catalytic core, Est2, binds centrally on the pseudoknot and utilises the template sequence (shown in red rectangle) to elongate telomeres. Est3 links Est1 and Est2, but does not directly bind Tlc1. The heterodimeric yKu70/80 binds to Tlc1 on the stem-loop IIc, while the Sm_7_ ring binds near the 3’ end of Tlc1. A schematic of the two-dimensional RNA structure prediction (without associated proteins) is depicted in the inset

To date, the yeast telomerase RNP is known to include Est1, Est2, Est3, Pop1, Pop6, Pop7, yKu70/80 and the Sm_7_ protein complexes. Investigations on the specific RNA-binding modes of many of those proteins have allowed to localise them on the predicted two-dimensional architecture of the Tlc1 RNA (Fig. 2; Dandjinou et al. 2004; Zappulla and Cech 2004; Lemieux et al. 2016). The Est1 and the three Pop proteins associate in close proximity on the IVc stem-loop (Fig. 2; Seto et al. 2002; Lemieux et al. 2016), Est2 on a proximal centre where three stems of the RNA are predicted to meet and form a pseudoknot (Livengood et al. 2002), and the yKu70/80 heterodimer binds to the apical 48 nt stem-loop located on the stem-loop IIc of Tlc1 (Fig. 2; Peterson et al. 2001; Stellwagen et al. 2003). Though Est3 is part of the telomerase RNP and is required for its in vivo activity, it does not directly bind Tlc1, instead acting as a linker between Est1 and Est2 (Fig. 2; Tucey and Lundblad 2014). Est2-Tlc1 form the minimal catalytic core of the enzyme, associating with Est1 and Est3 only at specific stages of the cell cycle, which may serve as a regulatory mechanism, restricting the time of telomerase function at telomeres (Fig. 1, step 5; Tucey and Lundblad 2014; Vasianovich et al. 2019). In addition, there is evidence of Est2 dissociation and telomerase disassembly after telomere elongation in late S/G2 phase (Tucey and Lundblad 2014). Est1 abundance appears to fluctuate during the cell cycle (Osterhage et al. 2006) which may restrict its ability to associate with Tlc1 to a specific window in late G1/S phase. Altogether, these events may help orchestrate the proper timing of telomerase competence for telomeres. In the absence of any of the Est proteins, the Tlc1 RNA tends to accumulate in the cytoplasm, suggesting the cytoplasmic association of Est subunits with the Tlc1 RNA (Gallardo et al. 2008; see below).

As mentioned above, the Pop1, Pop6 and Pop7 proteins associate with a P3-like stem on Tlc1 (IVc stem-loop), just distal to the Est1 binding site (Fig. 2; Lemieux et al. 2016; Laterreur et al. 2018). These proteins are also part of the evolutionarily conserved RNAse P/MRP complexes that are essential for cell viability (Esakova and Krasilnikov 2010). Furthermore, they have been implicated in ribosomal RNA pre-processing, tRNA processing and general cell cycle regulation in yeast (Esakova and Krasilnikov 2010). Pop1 acts as a protective scaffold for the Nme1 and Rpr1 RNAs in the RNAse P/MRP complexes by providing stabilisation for and shielding the P3 stem-loop, while attachment of Pop6 and Pop7 provide further support for Pop1 (Fagerlund et al. 2015). Likewise, the recruitment of Pop proteins to the Tlc1 P3-like stem could ensure correct architecture and provide protection prior to the complex acting on telomeres. Although cytoplasmic accumulation of Tlc1 has been reported in temperature-sensitive *pop1* and *pop6* mutants (Garcia et al. 2020), it is unclear if the accumulation is due to Tlc1 misfolding or pleiotropic effects associated with these temperature-sensitive mutations. Severe global metabolic disruptions brought on by temperature-sensitive alleles of essential genes could lead to grave cellular distress, and therefore expulsion of nuclear contents into the cytoplasm, eventually leading to cellular senescence. However, given that cytoplasmic accumulation of Tlc1 was also demonstrated for *est* mutants (Gallardo et al. 2008; Laterreur et al. 2013), one could propose that Tlc1 association with Pop1, Pop6 and Pop7, as well as the Est proteins occurs in the cytoplasm prior to nuclear import (Fig. 1, step 3). This would align with the idea that cytoplasmic telomerase RNP assembly is required for telomerase re-import into the nucleus and could serve as a checkpoint mechanism that prevents nuclear localisation of misfolded RNA and/or dysfunctional telomerase (Gallardo et al. 2008). Similarly, in *S. pombe,* the control of telomerase RNA architecture by the telomerase component Pof8 ensures that only correctly folded and therefore functional RNA can be utilised for telomere maintenance (Collopy et al. 2018; Mennie et al. 2018; Páez-Moscoso et al. 2018; Hu et al. 2020), further indicating the likely requirement for verification of proper cytoplasmic Tlc1 folding in *S. cerevisiae*.

The yKu heterodimer, comprised of yKu70 and yKu80, originally was discovered as the yeast homologue of human proteins associated with DNA end-binding in preparation for non-homologous end joining, a form of DNA double-strand break repair (Feldmann and Winnacker 1993; Boulton and Jackson 1996). Given that avoidance of end-to-end joining is a key function of telomeres, it was surprising that yKu proteins were associating with telomeric DNA ends (Gravel et al. 1998). Later work revealed that yKu also associated with the Tlc1 RNA and that DNA or RNA binding was exclusive to one or the other but yKu cannot simultaneously bind both (Peterson et al. 2001; Stellwagen et al. 2003; Pfingsten et al. 2012). It was reported that the yKu–telomerase interaction was important for short-lived telomerase–telomere encounters in G1 phase, supported by a concomitant yKu–Sir4 interaction (Fisher et al. 2004; Roy et al. 2004; Fisher and Zakian 2005; Chen et al. 2018). In addition, yKu was shown to associate with sub-telomeric sites where telomerase is not suspected to be and where it does not have access to chromosome ends. These associations have been proposed as an alternate method to ensure that the yKu–Sir4 interaction cannot enable unwanted telomere extensions during G1 (Bianchi et al. 2004; Larcher et al. 2016). This Sir4-mediated recruitment of telomerase to sites near telomeres may also serve to prevent telomerase association with DNA double-strand breaks, and thus block inappropriate healing of such sites by de novo telomere addition. Consistent with this idea, DNA break repair and telomere elongation appear to occur in different nuclear sub-compartments (Kramer and Haber 1993; Gartenberg 2009; Vasianovich et al. 2019). It must be noted that in principle, telomerase-mediated telomere elongation can occur in G1, but on normal telomeres, the presence of Rif1 and Rif2 prevents such events (Gallardo et al. 2011). Therefore, telomerase localisation to sub-telomeric sites via the Sir4–yKu–telomerase interaction appears not to be able to support telomere lengthening and at the same time keeps telomerase from reaching DNA double-strand breaks.

Telomerase normally only acts on telomeres in late S phase (Dionne and Wellinger 1996; Wellinger et al. 1996; Gallardo et al. 2011) and productive telomerase–telomere interactions during late S phase rely on an interaction between the telomere-binding protein Cdc13 and Est1 on telomerase (Evans and Lundblad 1999; Gallardo et al. 2011). Considering all the data together with the fact that in cells without the yKu–Sir4 interaction, only minor telomere length phenotypes are observed (Peterson et al. 2001), the functional relevance of the yKu–Sir4 pathway for telomerase recruitment in terms of telomere maintenance remains to be better defined.

Notwithstanding the above and similar to what was observed for cells harbouring mutations in *EST* genes, in cells expressing a *tlc1* allele that lacks the yKu binding site, the Tlc1 RNA appears to accumulate in the cytoplasm (Gallardo et al. 2008). This is consistent with a model predicting a yKu–Tlc1 RNA association in the cytoplasm, together with the other telomerase components (see above; Fig. 1, step 3). However, as discussed above for the Sm_7_ binding, it remains possible that the yKu–Tlc1 interaction occurs earlier, before the first nuclear export step. Newly developed techniques that permit the specific analyses of intracellular localisation of freshly transcribed versus matured Tlc1 RNAs will allow to pinpoint the correct interpretation of these early results (Vasianovich et al. 2020).

## Temporal succession of telomerase component associations and Tlc1 movement

In spite of the mentioned recent work showing how Tlc1 follows consecutive steps of maturation, i.e. first intranuclear stabilisation by Mex67/Mtr2, export to the cytoplasm by Xpo1, Sm_7_-mediated stabilisation in the cytoplasm, nuclear re-import, and Tgs1-dependent methylation in the nucleolus (Vasianovich et al. 2020), the question as to when precisely the Tlc1 RNA associates with the various proteins remains open. Some circumstantial evidence suggests that the Est, Pop and yKu associations occur in the cytoplasm before nuclear re-import and recruitment to telomeres. From a purely biophysical viewpoint, a complete telomerase RNP comprised of one Tlc1 RNA molecule complexed with Sm_7_, Est1/Est2/Est3, Pop1, Pop6/Pop7 and the yKu heterodimer should have a manageable size to be transported through nuclear pores into the nucleus. However, recently it has been demonstrated that as protein complexes grow larger, the more Nuclear Localisation Signal (NLS) units are required for nuclear import (Paci et al. 2020). Therefore, the predicted rather large size of the yeast telomerase of approximately 1,300 kDa (assumed from individual molecular weight predictions listed on https://www.rcsb.org/) likely causes a requirement for one or more NLSs for it to pass through the nuclear pore. These NLS sequences could be located on Est, yKu and/or Pop proteins, which, thus, would help to drive nuclear re-entry of the telomerase RNP (Fig. 1, step 4). Indeed, it would be of interest to determine the location and involvement of NLSs on the proteins of the telomerase RNP, and how their location may aid orientation and physical movement of the complex. On the other hand, if the number of required and accessible NLS domains is not suitable for nuclear import of the entire telomerase RNP, it brings to question whether some telomerase subunits may not be on the RNP at the time of re-import and associate later. For example, if the proposed cell cycle-dependent Est2 dissociation occurs in the nucleus, where will it reassociate? Furthermore, if the telomerase RNP cannot be transported as a fully assembled complex, this would suggest that stepwise binding of subunits could become a mechanistic checkpoint before telomerase is able to act on telomeres. This does not seem unreasonable due to the small number of fully assembled telomerase complexes and the importance of preventing unwarranted telomerase activity on non-telomeric sites.

Taken together, we speculate that the changing telomerase RNP composition could be a major reason for its biophysical behaviour in vivo. On the freshly made RNA, the assembled protein complexes may strongly favour its export to the cytoplasm, presumably mostly driven by the CBC, the TREX-1 and the Mex67/Mtr2 proteins (Fig. 1, step 3). Once in the cytoplasm, many of those proteins may dissociate from the RNA and new ones could assemble on it (for example the Sm_7_ ring; perhaps the yKu-, Est- and Pop proteins). This would cause a major change in the biochemical properties of the RNP and exposed NLSs could strongly favour nuclear re-entry of the RNP (Fig. 1, step 4). Hence, the interplay of nuclear import proteins and the accessible NLSs on telomerase subunits may become critical parameters that determine composition of the re-imported RNP. There may be additional changes once the RNP re-entered the nucleus, which could cause it to be exported again (e.g. temporal losses of Est proteins or of the yKu complex; Fig. 1, step 6). In essence, the specific composition of the RNP at any one point in time will strongly influence its biochemical properties and hence subcellular localisation. Therefore, the next challenge will be to determine the telomerase composition as it changes over time.

## Conclusions

The budding yeast telomerase RNA, Tlc1, is a large non-coding RNA that shares certain maturation and transport processes with snRNAs and mRNAs, but ultimately follows its own unique pathway. Despite the currently available information on Tlc1 transcription, modification and shuttling, many questions remain in regard to the full telomerase RNP lifecycle. What is the precise role of Mex67/Mtr2 on Tlc1 RNA after transcription? One possibility is that this complex acts as a chaperone and export adaptor, associating with newly transcribed Tlc1 and channelling it for Xpo1-mediated export, thereby preventing its degradation by the nuclear exosome (Vasianovich et al. 2020). This scenario raises additional questions: i.e. does Mex67/Mtr2 bind Tlc1 co-transcriptionally and is this association direct or mediated by other RNA-binding proteins, for example proteins of the TREX-1 complex? What other components of telomerase associate very early on the Tlc1 RNA? Is there indeed more than one shuttling event and what are the components responsible for shuttling rounds after the first one is complete? Clearly, the composition of the newly made, young telomerase RNP is different from the matured, or old RNP after one round of shuttling. How does this affect telomerase function and stability? Sub-cellular tracking of telomerase protein subunits alongside the Tlc1 RNA is a challenging prospect, but is a necessary endeavour for increasing the understanding of telomerase biogenesis and its complete lifecycle. Similar investigations are being performed on the human telomerase RNA as well and most importantly, there is very good evidence that these insights have clinical relevance (Vulliamy et al. 2006; Walne et al. 2007; Armanios and Blackburn 2012; Nagpal et al. 2020).

## Data Availability

Not applicable.

## References

[CR1] Amrani N, Sachs MS, Jacobson A (2006). Early nonsense: mRNA decay solves a translational problem. Nat Rev Mol Cell Bio.

[CR2] Armanios M, Blackburn EH (2012). The telomere syndromes. Nat Rev Gen.

[CR3] Bajon E, Laterreur N, Wellinger RJ (2015). A single templating RNA in yeast telomerase. Cell Rep.

[CR4] Becker D, Hirsch AG, Bender L, Lingner T, Salinas G, Krebber H (2019). Nuclear pre-snRNA export is an essential quality assurance mechanism for functional spliceosomes. Cell Rep.

[CR5] Bianchi A, Negrini S, Shore D (2004). Delivery of yeast telomerase to a DNA break depends on the recruitment functions of Cdc13 and Est1. Mol Cell.

[CR6] Bosoy D, Peng Y, Mian IS, Lue NF (2003). Conserved N-terminal motifs of telomerase reverse transcriptase required for ribonucleoprotein assembly in vivo. J Biol Chem.

[CR7] Boulon S, Verheggen C, Jady BE, Girard C, Pescia C, Paul C, Ospina JK, Kiss T, Matera AG, Bordonné R, Bertrand E (2004). PHAX and CRM1 are required sequentially to transport U3 snoRNA to nucleoli. Mol Cell.

[CR8] Boulton SJ, Jackson SP (1996). Identification of a *Saccharomyces cerevisiae* Ku80 homologue: roles in DNA double strand break rejoining and in telomeric maintenance. Nucleic Acids Res.

[CR9] Chan LY, Mugler CF, Heinrich S, Vallotton P, Weis K (2018). Non-invasive measurement of mRNA decay reveals translation initiation as the major determinant of mRNA stability. eLife.

[CR10] Chapon C, Cech TR, Zaug AJ (1997). Polyadenylation of telomerase RNA in budding yeast. RNA.

[CR11] Chatterjee K, Majumder S, Wan Y, Shah V, Wu J, Huang HY, Hopper AK (2017). Sharing the load: Mex67–Mtr2 cofunctions with Los1 in primary tRNA nuclear export. Genes Dev.

[CR12] Chen H, Xue J, Churikov D, Hass EP, Shi S, Lemon LD, Luciano P, Bertuch AA, Zappulla DC, Géli V, Wu J (2018). Structural insights into yeast telomerase recruitment to telomeres. Cell.

[CR13] Chen L, Roake CM, Galati A, Bavasso F, Micheli E, Saggio I, Schoeftner S, Cacchione S, Gatti M, Artandi SE, Raffa GD (2020). Loss of human TGS1 hypermethylase promotes increased telomerase rna and telomere elongation. Cell Rep.

[CR14] Cioce M, Lamond AI (2005). Cajal bodies: a long history of discovery. Annu Rev Cell Dev Biol.

[CR15] Collopy LC, Ware TL, Goncalves T, Í KongsstovuYang SQ, Amelina H, Pinder C, Alenazi A, Moiseeva V, Pearson SR, Armstrong CA, Tomita K (2018). LARP7 family proteins have conserved function in telomerase assembly. Nat Commun.

[CR16] Colot HV, Stutz F, Rosbash M (1996). The yeast splicing factor Mud13p is a commitment complex component and corresponds to CBP20, the small subunit of the nuclear cap-binding complex. Genes Dev.

[CR17] Dandjinou AT, Lévesque N, Larose S, Lucier JF, Abou Elela S, Wellinger RJ (2004). A phylogenetically based secondary structure for the yeast telomerase RNA. Curr Biol.

[CR18] Dias SM, Wilson KF, Rojas KS, Ambrosio AL, Cerione RA (2009). The molecular basis for the regulation of the cap-binding complex by the importins. Nat Struct Mol Biol.

[CR19] Diede SJ, Gottschling DE (1999). Telomerase-mediated telomere addition in vivo requires DNA primase and DNA polymerases α and δ. Cell.

[CR20] Dionne I, Wellinger RJ (1996). Cell cycle-regulated generation of single-stranded G-rich DNA in the absence of telomerase. Proc Natl Acad Sci USA.

[CR21] Dionne I, Wellinger RJ (1998). Processing of telomeric DNA ends requires the passage of a replication fork. Nucleic Acids Res.

[CR22] Dionne I, Larose S, Dandjinou AT, Abou Elela S, Wellinger RJ (2013). Cell cycle–dependent transcription factors control the expression of yeast telomerase RNA. RNA.

[CR23] Esakova O, Krasilnikov AS (2010). Of proteins and RNA: the RNase P/MRP family. RNA.

[CR24] Evans SK, Lundblad V (1999). Est1 and Cdc13 as comediators of telomerase access. Science.

[CR25] Fagerlund RD, Perederina A, Berezin I, Krasilnikov AS (2015). Footprinting analysis of interactions between the largest eukaryotic RNase P/MRP protein Pop1 and RNase P/MRP RNA components. RNA.

[CR26] Feldmann H, Winnacker EL (1993). A putative homologue of the human autoantigen Ku from *Saccharomyces cerevisiae*. J Biol Chem.

[CR27] Ferrezuelo F, Steiner B, Aldea M, Futcher B (2002). Biogenesis of yeast telomerase depends on the importin Mtr10. Mol Cell Biol.

[CR28] Fisher TS, Taggart AKP, Zakian VA (2004). Cell cycle-dependent regulation of yeast telomerase by Ku. Nat Struct Mol Biol.

[CR29] Fisher TS, Zakian VA (2005). Ku: a multifunctional protein involved in telomere maintenance. DNA Repair.

[CR30] Fortes P, Inada T, Preiss T, Hentze MW, Mattaj IW, Sachs AB (2000). The yeast nuclear cap binding complex can interact with translation factor eIF4G and mediate translation initiation. Mol Cell.

[CR31] Franke J, Gehlen J, Ehrenhofer-Murray AE (2008). Hypermethylation of yeast telomerase RNA by the snRNA and snoRNA methyltransferase Tgs1. J Cell Sci.

[CR32] Gallardo F, Olivier C, Dandjinou AT, Wellinger RJ, Chartrand P (2008). TLC1 RNA nucleo-cytoplasmic trafficking links telomerase biogenesis to its recruitment to telomeres. EMBO J.

[CR33] Gallardo F, Laterreur N, Cusanelli E, Ouenzar F, Querido E, Wellinger RJ, Chartrand P (2011). Live cell imaging of telomerase RNA dynamics reveals cell cycle-dependent clustering of telomerase at elongating telomeres. Mol Cell.

[CR34] Garcia PD, Leach RW, Wadsworth GM, Choudhary K, Li H, Aviran S, Kim HD, Zakian VA (2020). Stability and nuclear localization of yeast telomerase depend on protein components of RNase P/MRP. Nat Commun.

[CR35] Gartenberg MR (2009). Life on the edge: telomeres and persistent DNA breaks converge at the nuclear periphery. Gen Dev.

[CR36] Gingras AC, Raught B, Sonenberg N (1999). eIF4 initiation factors: effectors of mRNA recruitment to ribosomes and regulators of translation. Ann Rev Biochem.

[CR37] Görlich D, Kraft R, Kostka S, Vogel F, Hartmann E, Laskey RA, Mattaj IW, Izaurralde E (1996). Importin provides a link between nuclear protein import and U snRNA export. Cell.

[CR38] Gravel S, Larrivée M, Labrecque P, Wellinger RJ (1998). Yeast Ku as a regulator of chromosomal DNA end structure. Science.

[CR39] Greider CW, Blackburn EH (1985). Identification of a specific telomere terminal transferase activity in *Tetrahymena* extracts. Cell.

[CR40] Greider CW, Blackburn EH (1987). The telomere terminal transferase of *Tetrahymena* is a ribonucleoprotein enzyme with two kinds of primer specificity. Cell.

[CR41] Hamm J, Mattaj IW (1990). Monomethylated cap structures facilitate RNA export from the nucleus. Cell.

[CR42] Hu X, Kim JK, Yu C, Jun HI, Liu J, Sankaran B, Huang L, Qiao F (2020). Quality-control mechanism for telomerase RNA folding in the cell. Cell Rep.

[CR43] Huber J, Dickmanns A, Lührmann R (2002). The importin-β binding domain of snurportin1 is responsible for the Ran-and energy-independent nuclear import of spliceosomal U snRNPs in vitro. J Cell Biol.

[CR44] Hughes JMX, Konings DA, Cesareni G (1987). The yeast homologue of U3 snRNA. EMBO J.

[CR45] Hutten S, Kehlenbach RH (2007). CRM1-mediated nuclear export: to the pore and beyond. Trends Cell Biol.

[CR46] Izaurralde E, Lewis J, Gamberi C, Jarmolowski A, McGuigan C, Mattaj IW (1995). A cap-binding protein complex mediating U snRNA export. Nature.

[CR47] Jamonnak N, Creamer TJ, Darby MM, Schaughency P, Wheelan SJ, Corden JL (2011). Yeast Nrd1, Nab3, and Sen1 transcriptome-wide binding maps suggest multiple roles in post-transcriptional RNA processing. RNA.

[CR48] Jones MH, Guthrie C (1990). Unexpected flexibility in an evolutionarily conserved protein-RNA interaction: genetic analysis of the Sm binding site. EMBO J.

[CR49] Kramer KM, Haber JE (1993). New telomeres in yeast are initiated with a highly selected subset of TG1-3 repeats. Genes Dev.

[CR50] Kretzner L, Krol A, Rosbash M (1990). *Saccharomyces cerevisiae* U1 small nuclear RNA secondary structure contains both universal and yeast-specific domains. Proc Natl Acad Sci USA.

[CR51] Larcher MV, Pasquier E, MacDonald RS, Wellinger RJ (2016). Ku binding on telomeres occurs at sites distal from the physical chromosome ends. PLoS Genet.

[CR52] Larose S, Laterreur N, Ghazal G, Gagnon J, Wellinger RJ, Abou Elela S (2007). RNase III-dependent regulation of yeast telomerase. J Biol Chem.

[CR53] Laterreur N, Eschbach SH, Lafontaine DA, Wellinger RJ (2013). A new telomerase RNA element that is critical for telomere elongation. Nucleic Acids Res.

[CR54] Laterreur N, Lemieux B, Neumann H, Berger-Dancause JC, Lafontaine D, Wellinger RJ (2018). The yeast telomerase module for telomere recruitment requires a specific RNA architecture. RNA.

[CR55] Lemieux B, Laterreur N, Perederina A, Noël JF, Dubois ML, Krasilnikov AS, Wellinger RJ (2016). Active yeast telomerase shares subunits with ribonucleoproteins RNase P and RNase MRP. Cell.

[CR56] Lingner J, Cooper JP, Cech TR (1995). Telomerase and DNA end replication: no longer a lagging strand problem?. Science.

[CR57] Livengood AJ, Zaug AJ, Cech TR (2002). Essential regions of *Saccharomyces cerevisiae* telomerase RNA: separate elements for Est1p and Est2p interaction. Mol Cell Biol.

[CR58] Martin AA, Dionne I, Wellinger RJ, Holm C (2000). The function of DNA polymerase α at telomeric G tails is important for telomere homeostasis. Mol Cell Biol.

[CR59] Matera AG, Wang Z (2014). A day in the life of the spliceosome. Nat Rev Mol Cell Biol.

[CR61] Mennie AK, Moser BA, Nakamura TM (2018). LARP7-like protein Pof8 regulates telomerase assembly and poly(A)+ TERRA expression in fission yeast. Nat Commun.

[CR62] Mozdy AD, Cech TR (2006). Low abundance of telomerase in yeast: Implications for telomerase haploinsufficiency. RNA.

[CR63] Nagpal N, Wang J, Zeng J, Lo E, Moon DH, Luk K, Braun RO, Burroughs LM, Keel SB, Reilly C, Lindsley RC (2020). Small-molecule PAPD5 inhibitors restore telomerase activity in patient stem cells. Cell Stem Cell.

[CR64] Noël JF, Larose S, Elela SA, Wellinger RJ (2012). Budding yeast telomerase RNA transcription termination is dictated by the Nrd1/Nab3 non-coding RNA termination pathway. Nucleic Acids Res.

[CR65] Nostramo RT, Hopper AK (2020). A novel assay provides insight into tRNAPhe retrograde nuclear import and re-export in *S. cerevisiae*. Nucleic Acids Res.

[CR66] Ohno M, Segref A, Bachi A, Wilm M, Mattaj IW (2000). PHAX, a mediator of U snRNA nuclear export whose activity is regulated by phosphorylation. Cell.

[CR67] Okamura M, Inose H, Masuda S (2015). RNA export through the NPC in eukaryotes. Genes.

[CR68] Osterhage JL, Talley JM, Friedman KL (2006). Proteasome-dependent degradation of Est1p regulates the cell cycle–restricted assembly of telomerase in *Saccharomyces cerevisiae*. Nat Struct Mol Biol.

[CR69] Paci G, Zheng T, Caria J, Zilman A, Lemke EA (2020). Molecular determinants of large cargo transport into the nucleus. eLife.

[CR70] Páez-Moscoso DJ, Pan L, Sigauke RF, Schroeder MR, Tang W, Baumann P (2018). Pof8 is a La-related protein and a constitutive component of telomerase in fission yeast. Nat Commun.

[CR71] Palacios I, Hetzer M, Adam SA, Mattaj IW (1997). Nuclear import of U snRNPs requires importin β. EMBO J.

[CR72] Parenteau J, Wellinger RJ (2002). Differential processing of leading-and lagging-strand ends at *Saccharomyces cerevisiae* telomeres revealed by the absence of Rad27p nuclease. Genetics.

[CR73] Peña Á, Gewartowski K, Mroczek S, Cuéllar J, Szykowska A, Prokop A, Czarnocki-Cieciura M, Piwowarski J, Tous C, Aguilera A, Carrascosa JL (2012). Architecture and nucleic acids recognition mechanism of the THO complex, an mRNP assembly factor. EMBO J.

[CR74] Peterson SE, Stellwagen AE, Diede SJ, Singer MS, Haimberger ZW, Johnson CO, Tzoneva M, Gottschling DE (2001). The function of a stem-loop in telomerase RNA is linked to the DNA repair protein Ku. Nat Genet.

[CR75] Pfingsten JS, Goodrich KJ, Taabazuing C, Ouenzar F, Chartrand P, Cech TR (2012). Mutually exclusive binding of telomerase RNA and DNA by Ku alters telomerase recruitment model. Cell.

[CR76] Plessel G, Fischer U, Luhrmann R (1994). m3G cap hypermethylation of Ul small nuclear ribonucleoprotein (snRNP) in vitro: evidence that the Ul small nuclear RNA-(guanosine-N2)-methyltransferase is a non-snRNP cytoplasmic protein that requires a binding site on the Sm core domain. Mol Cell Biol.

[CR77] Podlevsky JD, Chen JJL (2016). Evolutionary perspectives of telomerase RNA structure and function. RNA Biol.

[CR78] Roithová A, Feketová Z, Vaňáčová Š, Staněk D (2020). DIS3L2 and LSm proteins are involved in the surveillance of Sm ring-deficient snRNAs. Nucleic Acids Res.

[CR79] Roy R, Meier B, McAinsh AD, Feldmann HM, Jackson SP (2004). Separation-of-function mutants of yeast Ku80 reveal a Yku80p-Sir4p interaction involved in telomeric silencing. J Biol Chem.

[CR80] Schwer B, Erdjument-Bromage H, Shuman S (2011). Composition of yeast snRNPs and snoRNPs in the absence of trimethylguanosine caps reveals nuclear cap binding protein as a gained U1 component implicated in the cold-sensitivity of *tgs1Δ* cells. Nucleic Acids Res.

[CR81] Seto AG, Zaug AJ, Sobel SG, Wolin SL, Cech TR (1999). *Saccharomyces cerevisiae* telomerase is an Sm small nuclear ribonucleoprotein particle. Nature.

[CR82] Seto AG, Livengood AJ, Tzfati Y, Blackburn EH, Cech TR (2002). A bulged stem tethers Est1p to telomerase RNA in budding yeast. Genes Dev.

[CR83] Singer MS, Gottschling DE (1994). TLC1: template RNA component of *Saccharomyces cerevisiae* telomerase. Science.

[CR84] Stanley GJ, Fassati A, Hoogenboom BW (2018). Atomic force microscopy reveals structural variability amongst nuclear pore complexes. Life Sci Alliance.

[CR85] Stellwagen AE, Haimberger ZW, Veatch JR, Gottschling DE (2003). Ku interacts with telomerase RNA to promote telomere addition at native and broken chromosome ends. Genes Dev.

[CR86] Stutz F, Liao XC, Rosbash M (1993). U1 small nuclear ribonucleoprotein particle-protein interactions are revealed in *Saccharomyces cerevisiae* by in vivo competition assays. Mol Cell Biol.

[CR87] Tang W, Kannan R, Blanchette M, Baumann P (2012). Telomerase RNA biogenesis involves sequential binding by Sm and Lsm complexes. Nature.

[CR88] Tucey TM, Lundblad V (2014). Regulated assembly and disassembly of the yeast telomerase quaternary complex. Genes Dev.

[CR89] Tudek A, Schmid M, Makaras M, Barrass JD, Beggs JD, Jensen TH (2018). A nuclear export block triggers the decay of newly synthesized polyadenylated RNA. Cell Rep.

[CR90] Vasianovich Y, Krallis A, Wellinger R, Morrish TA (2019). Telomerase in space and time: regulation of yeast telomerase function at telomeres and DNA breaks. Telomerase and non-telomerase mechanisms of telomere maintenance.

[CR91] Vasianovich Y, Bajon E, Wellinger RJ (2020). Telomerase biogenesis requires a novel Mex67 function and a cytoplasmic association with the Sm7 complex. eLife.

[CR92] Verheggen C, Lafontaine DLJ, Samarsky D, Mouaikel J, Blanchard JM, Bordonné R, Bertrand E (2002). Mammalian and yeast U3 snoRNPs are matured in specific and related nuclear compartments. EMBO J.

[CR93] Vulliamy TJ, Marrone A, Knight SW, Walne A, Mason PJ, Dokal I (2006). Mutations in dyskeratosis congenita: their impact on telomere length and the diversity of clinical presentation. Blood.

[CR94] Walne AJ, Vulliamy T, Marrone A, Beswick R, Kirwan M, Masunari Y, Al-Qurashi FH, Aljurf M, Dokal I (2007). Genetic heterogeneity in autosomal recessive dyskeratosis congenita with one subtype due to mutations in the telomerase-associated protein NOP10. Hum Mol Genet.

[CR95] Wan R, Yan C, Bai R, Wang L, Huang M, Wong CC, Shi Y (2016). The 3.8 Å structure of the U4/U6. U5 tri-snRNP: insights into spliceosome assembly and catalysis. Science.

[CR96] Wellinger RJ (2014). In the end, what’s the problem?. Mol Cell.

[CR97] Wellinger RJ, Ethier K, Labrecque P, Zakian VA (1996). Evidence for a new step in telomere maintenance. Cell.

[CR98] Wellinger RJ, Zakian VA (2012). Everything you ever wanted to know about *Saccharomyces cerevisiae* telomeres: beginning to end. Genetics.

[CR99] Zappulla DC, Cech TR (2004). Yeast telomerase RNA: a flexible scaffold for protein subunits. Proc Natl Acad Sci USA.

